# Reduction in bone relapse and improved survival with oral clodronate for adjuvant treatment of operable breast cancer [ISRCTN83688026]

**DOI:** 10.1186/bcr1384

**Published:** 2006-03-15

**Authors:** Trevor Powles, Alexander Paterson, Eugene McCloskey, Phil Schein, Bobbi Scheffler, Alwynne Tidy, Sue Ashley, Ian Smith, Lars Ottestad, John Kanis

**Affiliations:** 1 Parkside Oncology, London, UK; 2 Tom Baker Cancer Centre, Calgary, Alberta, Canada; 3 WHO Collaborating Centre for Metabolic Bone Diseases, University of Sheffield Medical School, Sheffield, UK; 4 Department of Clinical Pharmacology, University of Oxford, Oxford, UK; 5 Scheffler Group, Villanova, Pennsylvania, USA; 6 Royal Marsden Hospital, London, UK; 7 Radium Institute, Oslo, Norway

## Abstract

**Introduction:**

Experimental and clinical data show that the oral bisphosphonate clodronate (Bonefos^®^) can inhibit tumor-induced osteoclastic bone resorption. This randomized, double-blind, placebo-controlled, multicenter trial was designed to determine if the addition of oral clodronate to standard treatment for primary operable breast cancer could reduce the subsequent occurrence of bone metastases and thereby improve overall survival.

**Methods:**

1,069 patients with primary operable stage I-III breast cancer were randomized to receive oral clodronate (1,600 mg/day) or placebo for 2 years, in conjunction with standard treatment for primary breast cancer including surgery, radiotherapy, adjuvant chemotherapy, and/or tamoxifen. All patients were assessed for bone metastases at two and five years and additionally when clinically indicated. Survival status was determined as of the close of the study on 30 June 2000 with a median follow up of 5.6 years. The treatment arms were compared using the unstratified log-rank test. Hazard ratios (HRs) with 95% confidence intervals were calculated.

**Results:**

Oral clodronate significantly reduced the risk of bone metastases in all patients over the 5 year study period (51 patients versus 73 patients with placebo; HR = 0.692, *P *= 0.043); the difference was also statistically significant over the 2 year medication period (19 patients versus 35 patients with placebo; HR = 0.546, *P *= 0.031). These differences were most pronounced in patients with stage II/III disease (39 patients versus 64 patients with placebo, HR = 0.592, *P *= 0.009 over 5 years; 16 patients versus 32 patients with placebo, HR= 0.496, *P *= 0.020 over 2 years). Survival data also favoured the clodronate arm (HR for all patients = 0.768, *P *= 0.048; HR for stage II/III disease = 0.743, *P *= 0.041), although this was not significant due to multiple analyses. Oral clodronate was well tolerated, with mild-to-moderate diarrhoea being the most frequently reported adverse event.

**Conclusion:**

These results confirm that oral clodronate will significantly improve the 5 year bone relapse free survival when used as a supplementary adjuvant treatment for patients receiving standard treatment for primary operable breast cancer.

## Introduction

Adjuvant therapy of primary operable breast cancer using endocrine or chemotherapy represents a major advance in the treatment of this disease and has resulted in a significant improvement in relapse free and overall survival [1,2]. Bone is the most common distant site of recurrence for patients with breast cancer [3] and appears to be the site of metastases that gains least benefit from adjuvant chemotherapy [4].

Experimentally, anti-osteolytic agents including bisphosphonates have been shown to prevent the development of bone metastases in various animal models [5-9]. Bisphosphonates exert their anti-osteolytic effects by inhibiting osteoclast activity, and this mechanism is postulated to be the mechanism for prevention of experimental metastases [10,11]. Several placebo-controlled trials have reported that oral clodronate, oral ibandronate or intravenous pamidronate will reduce the skeletal complications in patients with metastatic breast cancer [12-15]. Oral clodronate has also been shown to reduce the incidence of bone metastases in women with advanced breast cancer [16] and in patients with primary breast cancer [17].

This randomized, double-blind, placebo-controlled study was designed to determine if oral clodronate 1,600 mg daily for 2 years in conjunction with standard adjuvant therapy could reduce the incidence of bone metastases in patients with primary, stageI-III breast cancer. Earlier analyses of this study were previously published [18,19]. We now report the final analyses of these data.

## Materials and methods

### Patients

This study population has been described previously. Briefly, all patients (*N* = 1,069) had histologically or cytologically confirmed primary operable breast cancer with no evidence of metastasis, and were recruited between 1989 and 1995. Primary treatment consisted of surgery and/or radiotherapy with or without systemic endocrine therapy and/or chemotherapy. Patients were excluded if they had significant renal, hepatic, or non-malignant bone disease; other exclusion criteria included a history of malignant disease or prior bisphosphonate use. All patients were required to be both psychologically and physically suitable for 2 years of treatment with oral clodronate or placebo. In accordance with the Declarations of Helsinki, all patients provided written informed consent prior to study participation.

### Study design

This randomized, double-blind, placebo-controlled, multinational, multicenter study designed to determine whether oral clodronate 1,600 mg daily for 2 years could reduce the occurrence of bone metastases over the 5 year study period. Eligible patients were stratified prior to randomization by center and by use of chemotherapy for pre-menopausal women. All patients were assessed for bone metastases at 2 years and 5 years and otherwise when clinically indicated. Clinical laboratory tests were repeated every 3 months during the first year of the study, and every 6 months during years 2 through 5. Patients were followed for survival until the study was formally closed on 30 June 2000 (median follow-up: 5.6 years; range 5 years to 10 years 6 months).

Each new patient was registered and randomized at each participating center by means of numerically ordered and coded packages containing either oral clodronate or placebo according to a centralized blinded code. Prior to randomization, the initial screening process included obtaining a clinical history, conducting a physical examination, assessing hematological, renal and hepatic function, determining urinary calcium, hydroxyproline and creatinine levels, and taking skeletal radiographs and bone scintigraphy. Other clinical investigations included computed tomography (CT) scanning and magnetic resonance imaging (MRI), if indicated. Radiological assessments for the detection of bone metastases were repeated at the end of the 2 year treatment period and again at the end of the 5 year study period (or at any other time, if clinically indicated).

At relapse, the study blind was maintained and patients received appropriate local or systemic therapies according to the treatment protocols of the individual study center. Study medication (oral clodronate or placebo) was discontinued if bone metastases occurred, and bisphosphonate therapy could be administered for treatment of bone metastases at the investigator's discretion.

### Study endpoints and evaluation

The primary efficacy endpoint was time to first bone metastases over the 5 year study period, and the secondary endpoints included overall survival and the occurrence of non-skeletal relapses. Bone metastases were diagnosed by isotopic bone scan, skeletal X-rays and CT or MRI if required. The final diagnosis of bone metastases and subsequent audits of the data were always performed blinded to the patient's study medication. The date of first bone relapse was determined as the date of the first test abnormality in patients subsequently confirmed as bone metastatic relapse. Vertebral collapse required confirmation of bone metastasis with MRI or CT. Non-osseous metastases were categorized as either soft-tissue metastases (for example, skin, lymph node, or other breast) or as visceral metastases (lung, pleura, liver, or central nervous system). The date of the first positive diagnostic test indicated the event date.

The incidence of adverse events and laboratory abnormalities were assessed every 3 months during the first year of treatment and every 6 months during years 2 to 5.

### Statistical methods

The target sample size of 1,000 patients was calculated to provide 95% power to detect a 25% reduction in bone metastases over 5 years. Efficacy endpoints were analyzed by unstratified log-rank statistic; hazard ratios (HRs) with 95% confidence intervals (CIs) were calculated using a Cox regression model with treatment as the only effect. As interim analyses using the O'Brien Flemming stopping rules had been conducted, to maintain an overall alpha level of 0.05, the final analyses presented herein are considered statistically significant if *P *≤ 0.048.

Comparability of treatment groups at baseline were compared using a multi-way ANOVA for continuous variables and logistic regression for categorical variables.

Primary analyses were conducted on the intent-to-treat population (1,069 patients). Analyses are also presented for subset of patients diagnosed with stage II/III disease as they are recognized to be at higher risk for metastases.

To address the issue regarding the possible compromise of treatment for patients with bone metastases by the previous use of adjuvant clodronate, we have examined the occurrence of metastatic bone events and mortality in the patients who developed bone metastases. These events included the requirement for osseous radiation therapy, the occurrences of metastatic fractures, hypercalcaemia spinal cord compression and the need for bone surgery. This was a retrospective analysis not included in the original protocol.

## Results

### Patient characteristics

Patient demographic and baseline clinical characteristics were almost identically matched between treatment groups (Table 1). The median patient age was 52.8 years and 52.7 years for the oral clodronate and placebo groups, respectively. Approximately 50% of the women in each treatment group were postmenopausal, and the majority of women (66% in the oral clodronate and 67% in the placebo groups) had stage II/III disease. The treatment groups were well balanced in terms of axillary lymph node involvement, hormone receptor status and adjuvant endocrine and chemotherapy (Table 1).

### Effect of clodronate and development of bone metastases and survival

**Figure 1:**
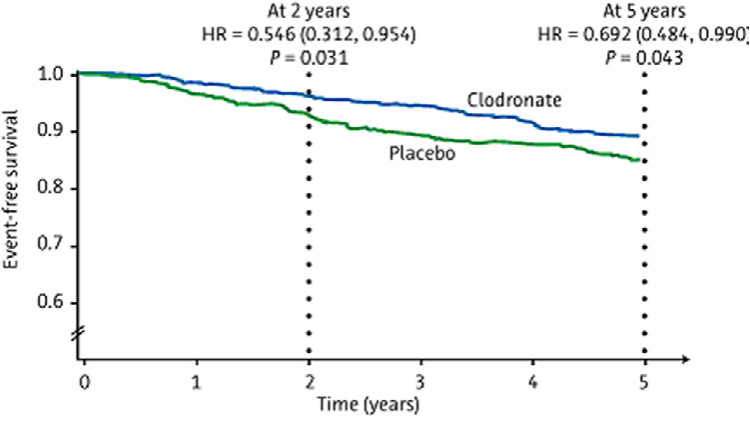
Incidence of bone metastases in all patients. HR, hazard ratio.

The addition of oral clodronate to adjuvant therapy for breast cancer significantly reduced the risk of bone metastases by 31% over the 5 year study period (51 patients versus 73 patients receiving placebo, HR = 0.692; *P *= 0.043; Figure 1 and Table 2). During the 2-year treatment period patients in the clodronate arm had a 45% reduction in the risk of bone metastases (*P *= 0.031; Table 2). Over the 5 year study period only 6% of patients with stage I disease developed bone metastases compared with 15% of patients with stage II disease and 34% of patients with stage III disease.

**Figure 2:**
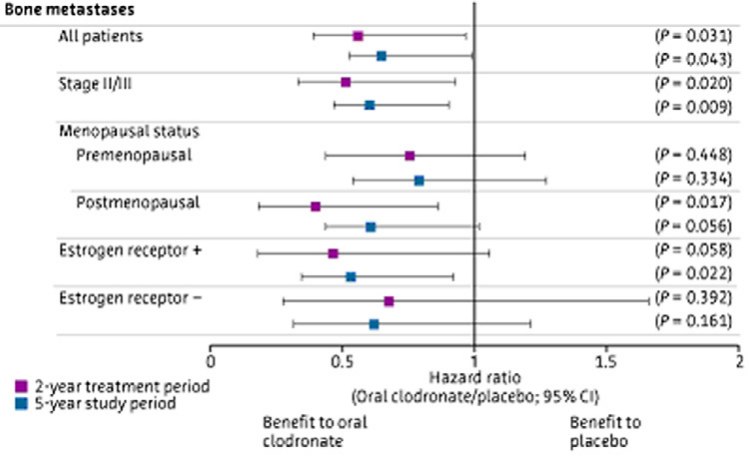
Hazard ratios and 95% confidence intervals (CI) for the incidence of bone metastases in patient subgroups.

**Figure 3:**
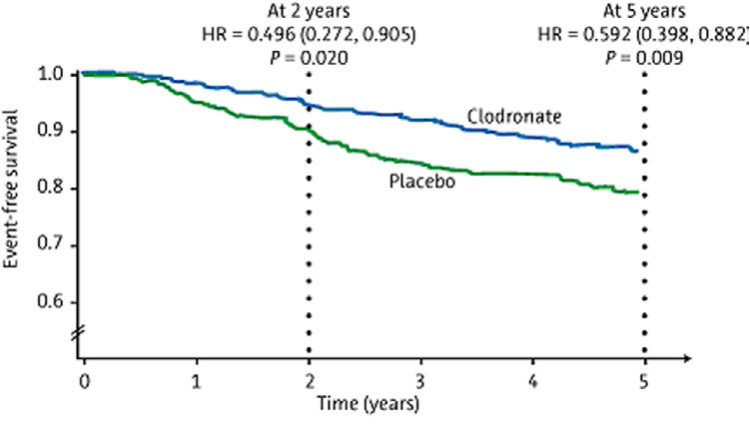
Incidence of bone metastases in stage II/III patients. HR, hazard ratio.

As shown in Figure 2 and Table 2, hazard ratios for bone metastases confirmed a similar risk reduction for bone metastases for all patient subgroups that received oral clodronate, apart from patients with relatively low risk stage I disease, where the numbers of events were too small to provide any meaningful comparison. For stage II/III disease, over the 5 year study period, the clodronate treated patients had a 41% decrease in the risk of developing bone metastases (*P *= 0.009); during the 2 year medication period, the risk was decreased by 50% (*P *= 0.020; Figure 3).

For the 51 of 530 clodronate patients and the 73 of 539 placebo patients who developed bone metastases, there was evidence of a reduction in the number of bone metastases-related bone events in clodronate patients (29/530; 5.5%) compared with placebo patients (53/539; 9.8%) (*P *< 0.01; Table 3). This shows that, although there were only 51 of the clodronate patients who relapsed in bone compared to 73 of the placebo patients, they did not do worse because of previous adjuvant clodronate. In fact, only 57% of these patients developed skeletal events compared to 73% of the bone relapsed placebo patients. This indicates that previous clodronate therapy does not appear to have compromised the subsequent treatment of relapsed metastatic bone disease.

With regard to non-osseous metastases arising during the 5 year study period, there was no significant difference in the occurrence of visceral metastases for patients randomized to clodronate: 79 patients versus 94 placebo patients (HR = 0.84, 95% CI 0.62–1.13; *P *= 0.24). For patients with stage II/III disease, there was a similar non-significant reduction in visceral metastases for women randomized to clodronate: 64 patients versus 81 placebo patients (HR = 0.77, 95% CI 0.56–1.07; *P *= 0.12; Table 2).

**Figure 4:**
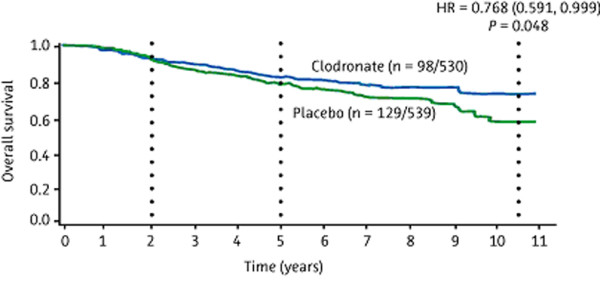
Overall survival in all patients. HR, hazard ratio.

**Figure 5:**
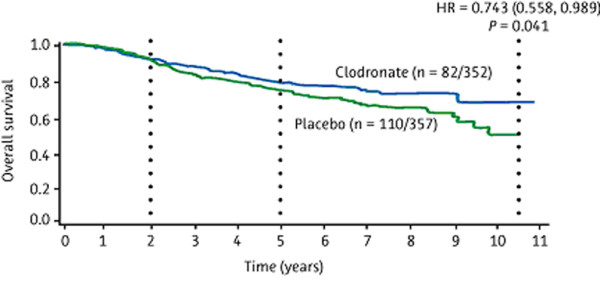
Overall survival in stage II/III patients. HR, hazard ratio.

Patients who received oral clodronate had an overall survival advantage as reported previously [18], although this is no longer statistically significant due to multiple analyses. This was observed for all patients (Figure 4) and for patients with stage II/III disease (Figure 5). There was a 23% reduction in the risk of death in all patients (HR = 0.768, 95% CI 0.591–0.999; *P *= 0.048) and a 26% reduction in risk of death in the subset of patients with stage II/III disease (HR = 0.743, 95% CI 0.558–0.989; *P *= 0.041), although neither of these reached statistical significance because of multiple analyses.

### Safety and tolerability

Overall, there were no reports of drug-related serious adverse events. Study medication was stopped early due to adverse events in 13% of patients receiving oral clodronate compared with 11% of patients receiving placebo.

Although the most frequently reported adverse events were gastrointestinal (Table 4), diarrhoea was the only adverse event to demonstrate a statistically significant increase in incidence in the clodronate treatment group. Diarrhoea occurred in 19.9% of the clodronate-treated patients versus 10% of the placebo-treated patients (*P *< 0.05) and was generally mild. There were no cases of oesophageal perforation or ulceration or osteonecrosis of the mandible and/or maxilla, which have been associated with the use of other bisphosphonates [20].

## Discussion

The addition of oral clodronate 1,600 mg daily for 2 years to standard adjuvant therapy significantly reduced the overall risk of bone metastases in patients with primary operable breast cancer during the first 5 years after primary diagnosis. The impact of oral clodronate therapy on bone metastasis was most pronounced during the 2 year treatment period but persisted throughout the 5 year study period.

Though not part of the original analysis plan, we examined the incidence of skeletal-related events in the patients who developed bone metastases. Treatment for these patients was at the discretion of the investigator. However, it was general practice to use a bisphosphonate when bone metastases occurred. Consistently, the data show fewer metastatic bone events in the patients who previously received adjuvant clodronate, supporting the benefit of adjuvant use rather than waiting until the bone metastases have occurred (Table 3). Furthermore, in this trial adjuvant clodronate did not show any evidence of a detrimental effect on visceral metastases and overall survival was significantly improved, clearly demonstrating that early treatment has clinical benefit.

The findings from this study confirm those of an earlier report of a small, randomized, open-label study with oral clodronate involving 302 women with newly diagnosed primary breast cancer, who also had micrometastases detected in the bone marrow [7,17]. These patients were randomly assigned to receive oral clodronate 1,600 mg/day or not during a 2 year treatment period. All patients received standard adjuvant therapies, including surgery, radiotherapy, endocrine therapy, and/or chemotherapy. The two arms were well balanced with respect to prognostic factors during the 3 year follow-up period. Patients who received oral clodronate had an approximately 50% reduction in the incidence of bone metastases (8% versus 17% with placebo; *P *= 0.003), and a significantly longer bone metastasis-free survival (*P *< 0.001) compared to those receiving standard treatment.

Another small, randomized, open-label study of similar size (N = 299) was published indicating no reduction in the incidence of skeletal metastases in the clodronate arm [21]. The investigators also claimed a negative effect of oral clodronate on visceral metastasis-free and overall survival, particularly in oestrogen receptor-negative breast cancer patients. There was, however, a significant imbalance in the treatment arms in this small, unblinded study, with respect to the important prognostic factors of oestrogen receptor status with significantly more oestrogen receptor-negative patients assigned to oral clodronate treatment (25 patients randomly assigned to clodronate treatment versus 10 patients to the control group; *P *= 0.03). Furthermore, these oestrogen receptor-negative patients were treated with endocrine therapy, rather than the established standard of chemotherapy for such patients [21]. Their reported significant reduction in survival was lost when this imbalance in oestrogen receptor negativity was corrected.

In contrast, our study reported here was a large, double blind, placebo-controlled trial. Randomization resulted in two treatment arms that were evenly balanced for all prognostic factors, including oestrogen receptor. Thus, the significant reduction in bone metastases and the trend towards improvement in overall survival observed in the clodronate arm in our trial we believe can be attributed to treatment with clodronate.

In the present study, oral clodronate was generally well tolerated. This safety profile is supported by over 260,000 patient years of marketing experience in 67 countries with clodronate used for treatment of hypercalcaemia and metastatic bone disease.

## Conclusion

The data from this trial indicate that the addition of oral clodronate, 1,600 mg daily for 2 years to standard adjuvant breast cancer therapy can significantly reduce the occurrence of bone metastases over a 5 year period. This benefit, together with the very low toxicity and substantial safety of clodronate, supports its use as additional adjuvant therapy for patients with primary breast cancer, particularly those at high risk of metastatic relapse. Questions such as the optional duration of therapy and efficacy in stage I disease remain to be answered.

## Abbreviations

CI = confidence interval; CT = computed tomography; HR = hazard ratio; MRI = magnetic resonance imaging.

## Competing interests

BS is a consultant for Berlex. TP is a lecturer/honoraria for Berlex. AHP is a consultant for Roche, Novartis, Berlex, Pfizer, Astrazeneca, Sopherion and Millenium. PS is a consultant for Berlex. J Kanis is a consultant for Schering. EM has recieved research funding & lecture fees from Schering. All other authors declare no competing interests.

## Authors' contributions

TP, principal investigator; AP, investigator; EMcC, investigator; PS, statistical, support; BS, statistical support; AT, principal data manager; SA, principal trial statistician; IS, investigator; LO, investigator; JK, investigator. All authors were involved in drafting and reviewing the manuscript.

## Figures and Tables

**Table 1:** Baseline demographics and clinical characteristics

Characteristic	Clodronate (*n* = 530)	Placebo (*n* = 539)
Mean age ± SD (years)	52.8 ± 10.6	52.7 ± 10.5
Mean BMI ± SD (kg/m^2^)	25.8 ± 4.5	25.8 ± 4.8
Menopausal status (*n*)		
Premenopausal	265 (50%)	265 (49%)
Postmenopausal	265 (50%)	274 (51%)
Axillary lymph node involvement (*n*)	196 (37%)	202 (38%)
Tumor stage (*n*)		
I	137 (26%)	143 (26%)
II	304 (57%)	305 (57%)
III	48 (9%)	52 (10%)
Unclassifiable	40 (8%)	39 (7%)
Missing	1 (0.2%)	0 (0%)
Hormone receptor status (*n*)		
Oestrogen receptor-positive	245 (46%)	240 (45%)
Oestrogen receptor-negative	136 (26%)	136 (25%)
Oestrogen receptor-unknown	149 (28%)	163 (30%)
Progesterone receptor-positive	112 (21%)	116 (22%)
Progesterone receptor-negative	79 (15%)	75 (14%)
Progesterone receptor-unknown	339 (64%)	348 (65%)

BMI, body mass index; SD, standard deviation.

**Table 2:** Incidence of bone metastases and mortality

	Clodronate (*n* = 530)	Placebo (*n* = 539)	Hazard ratio (95% CI)	*P *value
Bone metastases (all patients)				
2 years	19 (3.6%)	35 (6.5%)	0.546 (0.312, 0.954)	0.031
5 years	51 (9.6%)	73 (13.5%)	0.692 (0.484, 0.990)	0.043
Bone metastases (stage II/III)				
*n*	352	357		
2 years	16 (3.0%)	32 (6.0%)	0.496 (0.272, 0.975)	0.020
5 years	39 (7.4%)	64 (11.8%)	0.592 (0.398, 0.882)	0.009
Visceral metastases				
2 years	39 (7.3%)	47 (8.7%)	0.84 (0.55,1.29)	0.428
5 years	79 (14.9%)	94 (17.4%)	0.84 (0.62,1.13)	0.241
Visceral metastases (stage II/III)				
*n*	352	357		
2 years	34 (9.7%)	42 (11.8%)	0.81 (0.52,1.28)	0.372
5 years	64 (18.2%)	81 (22.7%)	0.77 (0.56,1.07)	0.120
Deaths during follow-up (median follow-up, 5.6 years)				
All patients	98 (18.5%)	129 (23.9%)	0.768 (0.591, 0.999)	0.048
Stage II/III	82 (23.3%)	110 (30.8%)	0.743 (0.558, 0.989)	0.041

CI, confidence interval.

**Table 3:** Skeletal-related events in patients developing bone metastases during the five year study period

	Clodronate	Placebo
Total number of patients	530	539
Skeletal-related event		
Confirmed bone metastases	51 (9.6%) % of bone	73 (13.5%) % of bone
Radiation therapy	24 (4.5%) (48%)	47 (8.7%) (69%)
Non-vertebral fracture	6 (1.1%) (2%)	16 (2.9%) (22%)
Vertebral fracture	6 (1.1%) (2%)	14 (2.6%) (19%)
Hypercalcemia	3 (0.5%) (1%)	11 (2.0%) (15%)
Spinal cord compression	2 (0.4%) (1%)	5 (0.9%) (7%)
Bone surgery	1 (0.2%) (1%)	4 (0.7%) (5%)
Overall for 5 year study period^1 ^(any skeletal event)	29 (5.5%) (0.4%)	53 (9.8%) (73%)

^1^*P *< 0.01 versus placebo.

**Table 4:** Incidence of gastrointestinal adverse events occurring in ≥ 10% of patients during the two year treatment period

	Percentage of patients with event	
	Clodronate (*n* = 530)	Placebo (*n* = 539)

Nausea	27.0	29.8
Diarrhoea^1^	19.9	10.0
Dyspepsia	15.6	13.3
Vomiting	14.7	13.1
Abdominal pain	13.6	10.5
Constipation	12.5	12.2
Stomatitis	8.4	10.2

^1^*P *< 0.05 versus placebo.
